# Human Motion Prediction via Dual-Attention and Multi-Granularity Temporal Convolutional Networks

**DOI:** 10.3390/s23125653

**Published:** 2023-06-16

**Authors:** Biaozhang Huang, Xinde Li

**Affiliations:** 1 Key Laboratory Measurement and Control of CSE Ministry of Education, School of Automation, Southeast University, Nanjing 210002, China; bzhuang@seu.edu.cn; 2 Nanjing Center for Applied Mathematics, Nanjing 211135, China

**Keywords:** human motion prediction, multi-granularity, attention mechanism, temporal convolutional networks

## Abstract

Intelligent devices, which significantly improve the quality of life and work efficiency, are now widely integrated into people’s daily lives and work. A precise understanding and analysis of human motion is essential for achieving harmonious coexistence and efficient interaction between intelligent devices and humans. However, existing human motion prediction methods often fail to fully exploit the dynamic spatial correlations and temporal dependencies inherent in motion sequence data, which leads to unsatisfactory prediction results. To address this issue, we proposed a novel human motion prediction method that utilizes dual-attention and multi-granularity temporal convolutional networks (DA-MgTCNs). Firstly, we designed a unique dual-attention (DA) model that combines joint attention and channel attention to extract spatial features from both joint and 3D coordinate dimensions. Next, we designed a multi-granularity temporal convolutional networks (MgTCNs) model with varying receptive fields to flexibly capture complex temporal dependencies. Finally, the experimental results from two benchmark datasets, Human3.6M and CMU-Mocap, demonstrated that our proposed method significantly outperformed other methods in both short-term and long-term prediction, thereby verifying the effectiveness of our algorithm.

## 1. Introduction

With the rapid development of artificial intelligence technology, an increasing number of intelligent devices are being applied in industrial production and daily human life. Human motion prediction, a key technology for enhancing device intelligence, aims to capture the intrinsic temporal evolution within historical human motion sequences to generate predictions for future motion. Human motion prediction has been widely applied in fields such as autonomous driving [1], human–computer interaction [2,3], human emotion recognition [4], and human behavior analysis [5,6,7]. However, due to the high dimensionality, joint spatial collaboration, hierarchical human structure, and strong temporality characteristics of human motion, capturing temporal dynamic information and spatial dependency features for precise human motion prediction remains a challenging research hotspot.

**Figure 1:**
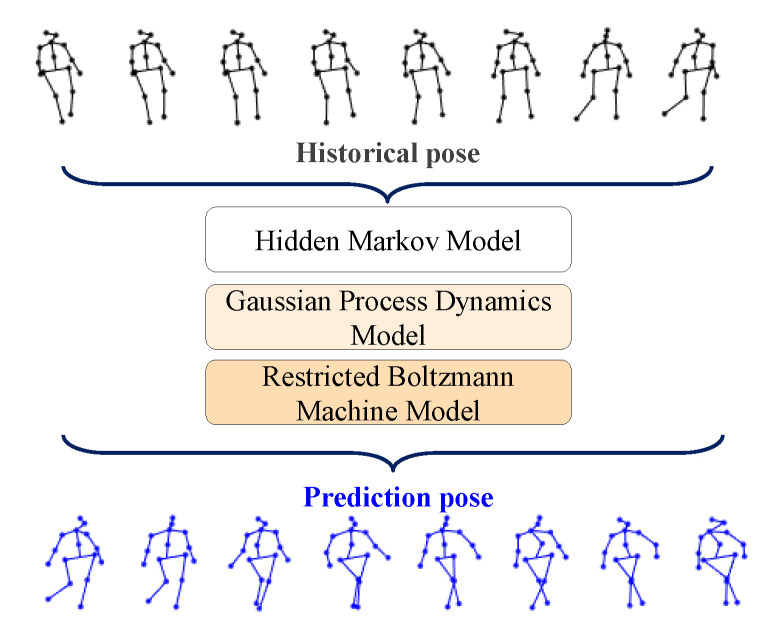
The process of human motion prediction. The top layer represents the historical pose data; the middle layer shows the classical methods used; and the bottom layer shows the output result, i.e., the predicted pose.

Human motion prediction is a typical task in the computer vision field. Traditional human motion prediction algorithms, such as hidden Markov models (HMMs) [8], Gaussian process dynamic models (GPDMs) [9], and restricted Boltzmann machines [10], as shown in Figure 1, often require extensive prior knowledge and assumptions, making it challenging to capture the complexity and diversity of human motion and so restricting their application impact.

As more and more large-scale motion capture datasets become available, an increasing number of deep learning models have been designed and have demonstrated excellent performance, such as convolutional neural networks (CNNs) [11], graph neural networks (GNNs) [12,13,14], temporal modules such as recurrent neural networks (RNNs) [15,16,17,18,19,20], temporal convolutional networks (TCNs) [21,22], and attention mechanisms [23,24]. Although these deep learning models have exhibited effectiveness in human motion prediction, there are still limitations in two aspects:

(a) Spatial relationship modeling: In most previous studies, spatial joint graphs were designed based on the human physical structure, typically utilizing graph neural networks (GNNs) [25] to capture spatial correlations. However, GNNs are limited by the local and linear aggregation of node features and may not effectively capture the global and nonlinear dynamics of human motion. The introduction of adaptive graphs aimed to overcome these limitations, but they still have drawbacks, such as overlooking the correlation between critical 3D coordinate information, which results in a loss of relevant internal data feature information.

(b) Simultaneously capturing complex short-term and long-term temporal dependencies: Most research has employed temporal learning components to capture temporal correlations. RNNs are a classic approach, but they face gradient vanishing or exploding issues when learning long time sequences. More advanced models such as LSTM and GRU mitigate the issue of vanishing gradients to a certain degree, but pose challenges in training and lack a parallel computation capability. Self-attention mechanisms [26,27] attempt to capture temporal dependencies but still struggle to effectively model long-range dependencies. TCNs [22,28] capture long-term dependencies through fixed kernel sizes, adopting an independent module framework that can only capture single dependency relationships from a temporal scale perspective. Fixed receptive fields limit their ability to adaptively learn multi-scale temporal dependencies.

To tackle these challenges in human motion prediction, a novel method based on dual attention and multi-granularity temporal convolutional networks (DA-MgTCNs) was proposed. This approach effectively captures spatial correlations and multi-scale temporal dependencies. Specifically, joint attention and channel attention were combined to design a dual-attention structure for extracting spatial features and capturing information on spatial correlations between and within human joints. TCNs were employed to model long-term temporal dependencies, and the concept of multi-granularity was introduced into the TCN to further enhance performance. The multi-granularity TCN (MgTCN) employed convolution kernels of varying scales in its convolution operations across multiple branches, enabling it to effectively capture multi-scale temporal dependencies in a flexible manner.

The MgTCN module was comprised of a combination of multi-granularity causal convolutions, dilated convolutions, and residual connections. Each branch of the module was composed of multiple causal convolution layers with varying dilation factors. This design enabled the adaptive selection of different receptive fields based on varying motion styles and joint trajectory features for short-term and long-term human motion prediction.

The main contributions of this paper are as follows:

(1) We designed a dual-attention model for extracting inter-joint and intra-joint spatial features, more effectively mining spatial relationships between joints and different motion styles, providing richer information sources for motion prediction.

(2) We introduced a multi-granularity temporal convolutional network (MgTCN) that employed multi-channel TCNs with different receptive fields for learning, thus achieving discriminative fusion at different time granularities, flexibly capturing complex short-term and long-term temporal dependencies, and thereby further improving the model’s performance.

(3) We conducted extensive experiments on the Human3.6M and CMU-MoCap datasets, demonstrating that our method outperformed most state-of-the-art approaches in short-term and long-term prediction, verifying the effectiveness of the proposed algorithm.

The remainder of this paper is organized as follows: Section 2 reviews related work. Section 3 details the proposed methodology. In Section 4, we describe experiments conducted on two large-scale datasets, comparing the performance of the proposed method with baselines. Section 5 provides a summary and conclusion, as well as a discussion of future work.

## 2. Related Work

In this section, we review the literature relevant to our dual-attention multi-granularity temporal convolutional networks (DA-MgTCNs) model, focusing on existing methods for human motion prediction, temporal convolutional networks (TCNs), multi-granularity (Mg) convolutions, and attention mechanisms.

### 2.1. Human Motion Prediction

The development of human motion prediction has evolved through several phases. Traditional methods primarily rely on statistical approaches, such as hidden Markov models (HMMs) [8], Gaussian processes (GPs) [9], and restricted Boltzmann machines [10], to learn underlying patterns and structures from data in order to predict future human motion [15]. Although these methods have achieved some success in certain scenarios, they still face challenges in capturing complex spatial and temporal dependencies, computational efficiency, and scalability.

With the rapid development of deep learning, researchers have started to apply it to human motion prediction tasks. Recurrent neural networks (RNNs) have been widely adopted for the temporal information modeling of human motion. Some representative works include Fragkiadaki et al. [15]’s RNN model, Martinez et al. [17]’s RNN-based joint angle prediction model, and Li et al. [11]’s convolutional recurrent neural network (CRNN) model. Although RNNs have achieved high accuracy in human motion prediction, they may lead to error accumulation and eventually converge to a statical average pose due to the continuous computation of time series.

To address this issue, researchers have improved RNNs. Chiu et al. [29] used LSTM units to model the underlying structure of human motion hierarchically, but this method did not adequately capture the spatial structure of the human body. Martinez et al. [17] introduced a residual structure using GRUs to model the velocity of human motion sequences, focusing on short-term temporal modeling but ignoring long-term dependencies and spatial structure. Jain et al. [16] combined LSTM and fully connected (FC) layers in a structural RNN model to encode high-level spatio-temporal structures in human motion sequences. Guo et al. [30] employed FC layers and GRUs to model local structures and capture long-term temporal dependencies, but they did not account for the interactions between different limbs. These RNN-based models faced challenges in capturing long-term dependencies and error accumulation.

### 2.2. Temporal Convolutional Networks

The temporal convolutional network (TCN) was developed to address these issues. The fundamental TCN architecture includes causal convolution, dilated convolution, and residual blocks [31]. Compared to RNNs and LSTMs, TCNs offer the advantages of parallel computation and larger receptive fields. Recent research has shown that 1D convolution can effectively represent time-series data [31,32,33], achieving significant success in various sequence learning tasks, such as machine translation [34], speech synthesis [35], video analysis [36], and semantic segmentation [37]. The contextual size of the network may be easily increased by stacking numerous one-dimensional convolutional layers, and creating hierarchical feature representations for input sequences enables the efficient modeling of long-term temporal patterns [35,36].

### 2.3. Multi-Granularity Convolution

A single-scale TCN might not be sufficient to capture the multi-scale temporal correlations in motion sequences in human motion prediction tasks. To capture complicated short-term and long-term temporal connections, researchers have developed a method known as multi-granularity convolution, which can combines multi-scale information fusion [38]. By adjusting the convolutional size, CNN-based deep learning models can quickly gather feature information at various granularities, enabling more accurate decision making by combining and evaluating data from various scales. Recent achievements in the field of computer vision have fully exploited multi-granularity information fusion based on CNNs [39].

### 2.4. Attention Mechanisms

Additionally, understanding spatial relationships is essential when attempting to predict human motion. In order to overcome this constraint, attention mechanisms were incorporated into the model to extract the spatial correlations of joints. There is still untapped potential in the area of human motion prediction, despite the widespread use of attention mechanisms in natural language processing [40,41] and image processing [42]. Tang et al. [43] employed the attention module for information extraction along the temporal dimension, and Cai et al. [44] used it for global spatial dependency among joint trajectories. However, it is believed that the intrinsic three-dimensional coordinate information of the human body is crucial for spatial representation.

## 3. Approach

### 3.1. Problem Formulation

Our goal was to forecast future human posture sequences based on previous 3D human pose sequences. Three-dimensional joint positions were employed as the pose representation to prevent the ambiguity produced by the joint angle representation. A graphical representation of the human pose was created by analysing the properties of human joint positions over time. Let $x_{1:T}=\left[x_{1},x_{2},\ldots ,x_{T}\right]$ represent the set of joint positions for *T* time steps, where $x_{i}\in R^{J\times C}$, *T* specifies the number of input time steps; *J* the number of human pose joints; and C=3 the feature dimension (x, y, z). Our goal was to anticipate the pose’s future steps $x_{T+1:T+N}=\left[x_{T+1},x_{T+2},\ldots ,x_{T+N}\right]$. We began by copying the latest pose $x_{T}N$ times to build a time series of length T+N, as described in the literature [25,45]. As a result, the goal became generating a time series of length T+N from the input sequence $x_{1:T+N}=\left[x_{1},x_{2},\ldots ,x_{T};x_{T},\ldots ,x_{T}\right]$ to produce the output sequence ${\hat{x}}_{1:T+N}$, where $x_{i}$ is commonly designated as the 3D coordinates of *N* body joints.

### 3.2. Overview

We employed a residual depth network consisting of DA-MgTCN modules to capture the global spatial correlation and multi-scale temporal dependence of human motion. Each DA-MgTCN module consisted of a two-branch attention structure module (DA) and a multi-granularity TCN module (MgTCN) connected in series to capture the inter-temporal dependence of historical motion sequences. The DA module was used to extract spatially significant information from joint-level and channel-level dimensions. A combination of multi-granularity convolution and TCN was used in the MgTCN module to increase the prediction quality and adapt to varied forms of human motion and multi-scale time. The complete model architecture was trained end-to-end, with global and local residual connections improving the deep neural network’s performance. Each DA-MgTCN component is described in detail below.

(1) $$Y_{DA-MgTCN}=MgTCN\left(DA\left(X\right)\right)$$

**Figure 2:**
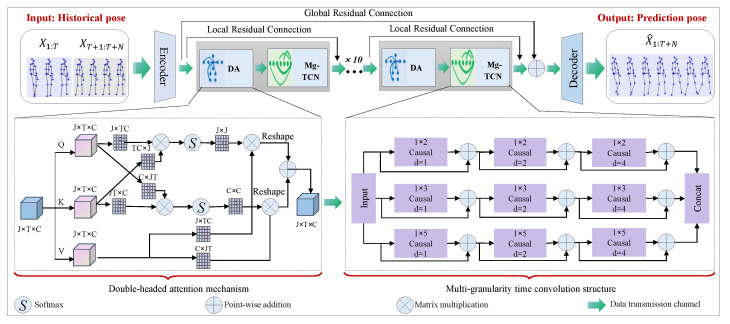
The whole architecture of our suggested solution for motion prediction, which employed an end-to-end framework. We encoded human poses $X_{1:T+N}$ and fed them into the DA-MgTCN, which was a series connection of DA and MgTCN modules. The DA module was used to extract spatially important information from dimensions at the joint and channel levels. The MgTCN was used to capture different scales of temporal dependencies. Finally, the decoder module recovered the time dimension length.

Figure 2 shows a detailed description of the module. The specifics of the DA and MgTCN modules are provided below.

### 3.3. Dual Attention (DA)

The self-attentive mechanism is regarded as an efficient method for modeling remote dependencies. Tang et al. [43] and Cai et al. [44] used the attention module for information extraction along the temporal dimension and the modeling of global spatial dependencies, respectively. However, we observed that the 3D coordinate information from human joints is crucial for spatial representations.

As a result, we proposed a dual-attention module that took into account both joint-level attention and channel-level attention in order to extract joint-related and channel-related information for spatial correlation. The DA module is depicted in the lower left corner of Figure 2 and is described in detail below.

Given a human motion feature *X*, a linear transformation was first performed using the weight matrices $W^{q}$, $W^{k}$, and $W^{v}$ to obtain the query *Q*, the key *K*, and the value *V*. These two branches shared the same embeddings: *Q*, *K*, and *V*. The embeddings are partially reorganized into J×CT (for *Q*, *K*, and *V* in the joint branch) and C×JT (for *Q*, *K*, and *V* in the channel branch) dimensions. The joint and channel attention were used to simultaneously mine the dependencies between joints in the space and channel dimensions. This was computed as follows:

(2) $$\left\{\begin{array}{c} Q=W^{q}X,\\ K=W^{k}X,\\ V=W^{v}X \end{array}\right.$$

(3) $$\begin{array}{cc} F_{J} & =Attention(Q_{\left(J\right)},K_{\left(J\right)},V_{\left(J\right)})\\ \; & =softmax\left(\frac{Q_{\left(J\right)}K_{\left(J\right)}^{T}}{\sqrt{d_{k}}}\right)V_{\left(J\right)} \end{array}$$

(4) $$\begin{array}{cc} F_{C} & =Attention(Q_{\left(C\right)},K_{\left(C\right)},V_{\left(C\right)})\\ \; & =softmax\left(\frac{Q_{\left(C\right)}K_{\left(C\right)}^{T}}{\sqrt{d_{k}}}\right)V_{\left(C\right)} \end{array}$$

where $Q_{\left(J\right)},K_{\left(J\right)},V_{\left(J\right)},Q_{\left(C\right)},K_{\left(C\right)},$ and $V_{\left(C\right)}$ represent the deformations of the Q,K, and *V* matrices, respectively. $W^{q}$, $W^{k}$, and $W^{v}$ are trainable weights, and $d_{k}$ is the dimension of K. J and C denote joint-level and channel-level branches, respectively. $F_{J}$ and $F_{C}$ are the output features of the joint-level and channel-level networks. After obtaining the joint-level and channel-level features, we summed them one by one to obtain the spatially noticed feature representation $\hat{X}$ of the MgTCN, as shown in Equation (5):

(5) $$\hat{X}=F_{J}⊕F_{C}$$

After obtaining the spatially noticed feature representation $\hat{X}$ of the motion data, we could feed this representation into the subsequent layers of the network. This process helped to capture joint-level and channel-level contextual information, which is crucial for effective motion prediction modeling.

### 3.4. Multi-Granularity TCN (MgTCN)

To learn human motion temporal features efficiently, we extended the concept of temporal multi-granularity convolutional kernels to TCN networks and proposed MgTCNs for extracting temporal features at multiple scales for different motion styles. The MgTCN module is shown in the lower right corner of Figure 2 and consisted of multi-granularity causal convolution, dilated convolution, and residual blocks. There were three causal convolution channels in the MgTCN, each using kernels with granularity sizes of 2, 3, and 5 for feature extraction. Each channel consisted of three residual blocks connected in series. These units increased the perceptual field at a dilation rate of [1, 2, 4] and used ReLU as the activation function. In addition, a dropout unit was included in each residual block for regularization.

Causal convolution: The output at the tth timestamp for standard 1D convolution is calculated from the k elements around the previous layer with time step t, which is not reasonable for the human motion prediction task [31]. The goal of this research was to find the best function for generating human-like future poses based on previous motion capture sequences. As a result, the predicted pose at time step t could be derived only from all possible representations of previously observed frames and not from later poses. MgTCN’s causal convolution ensured that only past data were used as the model input, preventing future information leakage. This was easily accomplished by shifting the standard convolution output by a few time steps, as shown in the equation below:

(6) $$y\left[t\right]=\sum_{i=0}^{k}x\left[t-i\right]*w\left[i\right]$$

where y[t] is the output, x[t−i] are the inputs, w[i] is the convolution weight at time step *i*, and *k* is the kernel size.

Dilated convolution: Causal convolution captures historical data inadequately. Increasing the network’s depth or number of layers can help it capture historical data linearly. However, increasing the network depth exponentially increases the number of parameters, making network training more difficult. Oord et al. [35] suggested using dilation convolution to extend causal convolutional networks’ receptive field to better capture historical information.

Dilated convolution is implemented by adding expansion parameters to the moving convolutional kernel. Compared to traditional deep convolutional networks, dilation convolution can obtain a larger receptive field without significantly increasing the number of parameters, thus capturing information over a longer time range. This approach can focus on both local details and motion trends over a longer time span when dealing with human motion prediction tasks.

Dilated causal convolution can be expressed by the following equation. For a filter f=(0,…,k−1) and $x\in R^{T}$, denoting the given 1D time-series input, the dilated convolution operation *F* on element *s* of the sequence is computed as:

(7) $$F_{k,d}\left(x\right)=\sum_{i=0}^{k-1}f\left(i\right)\cdot x\left(s-d\cdot i\right)$$

where *d* is the dilation factor, *k* is the size of the filter, and the convolution kernel is restricted to slide only at the current position and to its left (i.e., past information).

The receptive field *R* for a three-layer convolution is calculated as:

(8) $$R=1+\left(k-1\right)\times d_{1}+\left(k-1\right)\times d_{2}+\left(k-1\right)\times d_{3}$$

where $d_{1}$, $d_{2}$, and $d_{3}$ are the dilation factors of the three-layer convolution, which are used to calculate the size of the receptive field.

Our TCN was calculated as:

(9) $$\left\{\begin{array}{c} TCN_{k,d=1}=x+F_{k,d=1}\left(x\right),\\ TCN_{k,d=2}=TCN_{k,d=1}+F_{k,d=2}\left(TCN_{k,d=1}\right),\left(k=2,3,5\right)\\ TCN_{k}=TCN_{k,d=4}=TCN_{k,d=2}+F_{k,d=4}\left(TCN_{k,d=2}\right) \end{array}\right.$$

**Figure 3:**
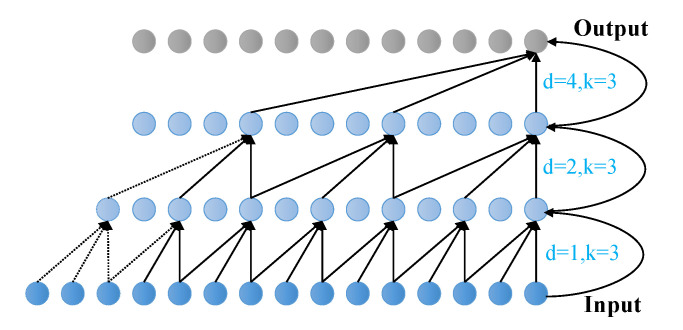
Architectural elements in a TCN (causal dilated convolutional network).

Figure 3 shows an example with a three-layer causal expansion convolutional network (TCN). The TCN’s elements in Figure 3 include a series of dilation causal convolutions with dilation factors d=1,2,4 and a filter size K=3.

Multi-granularity convolution: In order to handle complex, multi-action, multi-joint predictions of the human body, MgTCN required the use of convolutional kernel filters with different granularities to extract time-series features at different scales. This was necessary to meet the needs of short- and long-term predictions that require the capture of time-series features of different lengths. Three MgTCN time series were processed separately, which made it possible to combine multiple time granularities in the feature extraction process, which could better represent a large range of spatio-temporal features. Therefore, integrating time-series data with different time granularities to obtain better results is a challenge.

In order to handle the complex multi-action and multi-joint prediction of the human body, one must integrate time-series data with different time granularities to obtain better results. MgTCN used convolutional kernel filters with different granularities to extract time-series features at different scales. This satisfied the need for capturing short- and long-term forecasts of time-series features of different lengths. Three time series were treated separately in MgTCN, which allowed us to combine multiple temporal granularities in the feature extraction process to better represent certain large ranges of spatio-temporal features.

The MgTCN network output could be used to extract multi-temporal granularity features (short-term and long-term) using the aforementioned spatial and temporal feature extraction steps. We combined the data from these three TCN channels and used the equation below to make predictions in order to achieve the integration of the multi-granularity information.

(10) $$\begin{array}{cc} Fusion & =Cat(w_{1}\times TCN_{k=2},w_{2}\times TCN_{k=3},w_{3}\times TCN_{k=5}) \end{array}$$

(11) $$\begin{array}{cc} MgTCN & =g(Fusion) \end{array}$$

where $w_{i}$ is a learnable parameter to adjust the weights for different time periods, and g(.) represents a mapping function that maps the fused features to the predicted values.

With this multi-granularity temporal convolution (MgTCN) method, we could both observe the general trend of human motion in the long-term and capture the outliers of short-term changes. This temporal correlation facilitated predictive power.

### 3.5. Global and Local Residual Connection

Residual connection skips a layer of the network and adds its output to the next layer’s output. This eliminates gradient fading by propagating the gradient straight from the back layer to the front layer. This architecture simplifies neural network representation learning in deeper structures.

Figure 2 illustrates the use of global residual connections between the encoder and decoder modules and local residual connections in each DA-MgTCN module to enhance neural network training and deeper structural performance. This method assisted the network in capturing complex data patterns in human motion prediction.

### 3.6. Loss Function

To train our DA-MgTCN model, we employed an end-to-end training technique. The mean position per joint error (MPJPE) loss function between the anticipated motion sequence and the ground truth motion sequence was used to analyze the difference between the predicted outcomes and the true pose, which was defined as follows:

(12) $$L=\frac{1}{N}\sum_{i=1}^{N}\sum_{j=1}^{T}\left|\right|{\hat{Y}}_{i,j}-Y_{i,j}{\left|\right|}_{2}^{2}$$

where *N* is the number of human joints, *T* is the number of time steps in the future series, ${\hat{Y}}_{i,j}\in R^{C}$ is the prediction of the *i*th joint at the *j*th time step, and $Y_{i,j}$ is the corresponding ground truth.

We optimized the loss function using the improved Adam method (AdamW [46]), which mitigated the overfitting problem by adding a weight decay term and could significantly improve the robustness of the model.

## 4. Experiments

In this section, we evaluate the performance of the proposed method using two large-scale human motion capture benchmark datasets: Human3.6M and CMU-Mocap.

### 4.1. Datasets

Human3.6M [47] is the largest existing human motion analysis database, consisting of 7 actors (S1, S5, S6, S7, S8, S9, and S11) performing 15 actions: walking, eating, smoking, discussing, directions, greeting, phoning, posing, purchases, sitting, sitting Down, taking photos, waiting, walking a dog, and walking together. Some actions are periodic, such as walking, while others are non-periodic, such as taking photos. Each pose includes 32 joints, represented in the form of an exponential map. By converting these into 3D coordinates, eliminating redundant joints, global rotation, and translation, the resulting skeleton retains 17 joints that provide sufficient human motion details. These joints include key ones that locate major body parts (e.g., shoulders, knees, and elbows). This strategy ensures that no crucial joints are overlooked. We downsampled the frame rate to 25 fps and used S5 and S11 for testing and validation, while the remaining five actors were used for training.

CMU-MoCap, available at http://mocap.cs.cmu.edu/, accessed on 13 June 2023, is a 3D human motion dataset released by Carnegie Mellon University that used 12 Vicon infrared MX-40 cameras to record the positions of 41 sensors attached to the human body, describing human motion. The dataset can be divided into six motion themes, including human interaction, interaction with environment, locomotion, physical activities and sports, situations and scenarios, and test motions.

These motion themes can be further subdivided into 23 sub-motion themes. The same data preprocessing method as in the literature [25] was adopted, simplifying each human body and reducing the motion rate to 25 frames per second. Furthermore, eight actions (basketball, basketball signals, directing traffic, jumping, running, soccer, walking, and washing the face) were selected from the dataset to evaluate the model’s performance. No hyperparameters were adjusted in this dataset, and we only used the training and testing sets, applying a splitting method consistent with the common practice in the literature.

### 4.2. Implementation Details

All experiments in this paper were implemented using the PyTorch deep learning framework. The experimental environment was Ubuntu 20.04 with an NVIDIA A100 GPU. During the training process, the batch normalization size was set to 16, and the AdamW optimizer was used to optimize the model. The initial learning rate was set to 0.003, with decay by 5% every 5 epochs. The model was trained for 60 epochs, and each experiment was conducted three times. The average result was taken to ensure a more robust evaluation of the model’s performance. The input motion prediction length was 25 frames (1000 ms), and the prediction generated 25 frames (1000 ms). The choice and configuration of the relevant hyperparameters are shown in Table 1.

### 4.3. Evaluation Metrics and Baselines

The same evaluation metrics as those used in existing algorithms [25,45] were employed for assessing model performance. The standard mean per joint position error (MPJPE) was used to measure the average Euclidean distance (in millimeters, mm) between the predicted joint 3D coordinates and the ground truth, as illustrated in Equation (12). In addition, to further illustrate the advantages of the method, we conducted a comparative analysis of our method with Res. sup. [17], convSeq2Seq [11], DMGNN [13], LTD [25], LPJP [44], Hisrep [48], MSR [49], and ST-DGCN [45].

### 4.4. Experimental Results and Analysis

**Human3.6M**: Based on the existing work, we divided the prediction results into short-term (80–400 ms) and long-term predictions (500–1000 ms). The experimental results are shown in Table 2, which demonstrates the joint position error and mean error for short-term (80 ms, 160 ms, 320 ms, 400 ms) and long-term (560 ms, 1000 ms) predictions for 15 kinds of movements. It was found that the existing methods usually showed high prediction accuracy when dealing with more periodic and regular movements, such as “walking” and “eating”. However, when dealing with more random and irregular movements, such as “directions”, “posing”, and “purchases”, the prediction accuracy decreased significantly. The algorithm proposed in this paper showed high prediction accuracy when dealing with highly complex, non-periodic, and irregular movements.

Our experimental results revealed that the proposed DA-MgTCN method outperformed most baseline methods in both short-term and long-term motion prediction. In particular, it can be observed from the experimental results that the proposed DA-MgTCN method outperformed most baseline methods in short-term motion prediction and improved more significantly in long-term prediction, with each MPJPE index reaching the optimum and obtaining excellent prediction results for both the 560 mm and 1000 mm MPJPE metrics. This success can be attributed to the ability of DA-MgTCN to fully capture spatial correlation and multi-granularity temporal features, which was a key factor in enhancing the model’s prediction accuracy.

**Figure 4:**
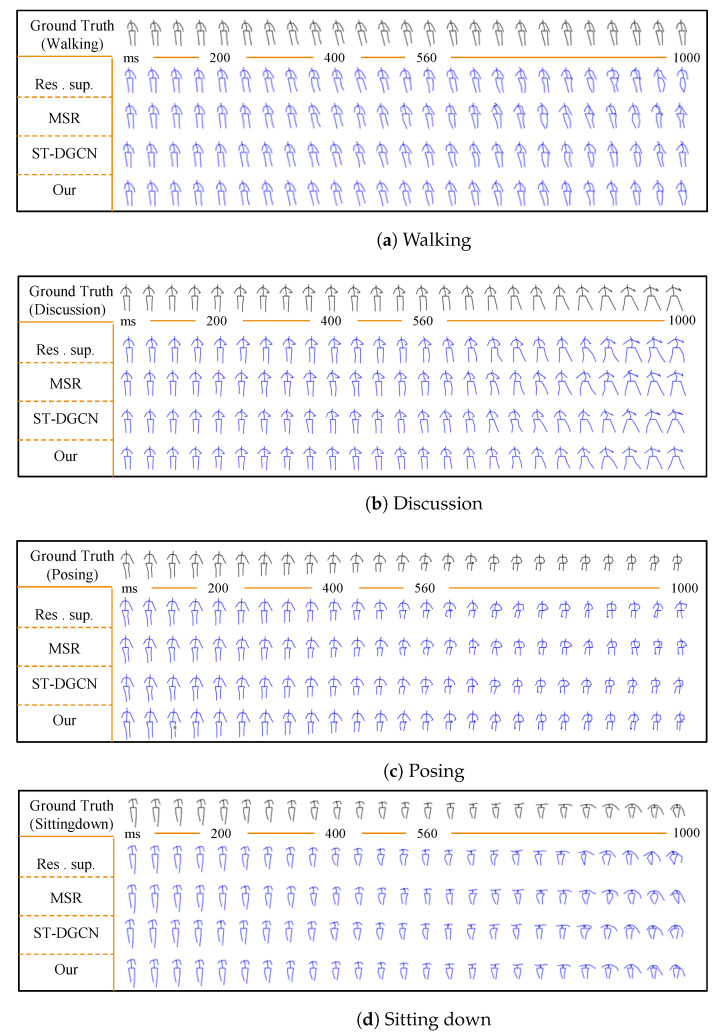
Qualitative comparison.

Qualitative comparison: We visualized the results of the aforementioned motion prediction to further assess the model’s performance. Figure 4 illustrates the visualization results for actions including “walking”, “discussion”, “posing”, and “sitting down”. The first row in every subplot shows the ground truth pose sequences (in black), followed by the predicted poses (in blue), i.e., each row displays the prediction results of one model. From the visualization results, it was observed that the predictions generated by the DA-MgTCN method showed higher similarity to the actual sequences and exhibited lower distortion and better continuity between frames. This was due to the dual-branch spatial attention and multi-granularity temporal convolution modeling joint motion trajectories, which provided richer and smoother joint motion temporal context information. The model could sufficiently capture global spatial dependencies, allowing it to encode joint information with distant hidden dependencies. For example, in the “sitting down” motion visualization, the motion between the hands and feet was more coordinated and coherent. This demonstrated once again how well the suggested DA-MgTCN forecasted very complicated irregular movements and complex periodic motions.

**CMU-MoCap:** To further validate the generalization of the DA-MgTCN method, we compared its performance with existing algorithms on the CMU-MoCap dataset, including Res. sup. [17], convSeq2Seq [11], DMGNN [13], LTD [25], LPJP [44], MSR [49], and ST-DGCN [45]. The experimental results are shown in Table 3, presenting the mean per joint position error and corresponding average error for short-term and long-term predictions across eight actions. From the table, it can be observed that the DA-MgTCN method’s short-term and long-term prediction accuracy was significantly higher than that of the other seven existing prediction algorithms, including Cai et al. [44]’s method, even when handling relatively complex non-periodic actions. The DA-MgTCN method improved the average prediction accuracy by about 1.5% in short-term prediction and 3% in long-term prediction, respectively, compared to the state-of-the-art ST-DGCN method. Thus, the comprehensive experimental results once again confirmed the effectiveness and generalization capabilities of the DA-MgTCN method.

### 4.5. Ablation Study

To deeply evaluate the contribution of each component in our model, we conducted a series of ablation experiments on the Human3.6M dataset. These experiments focused on the impact of the channel-attention (channel-att) and multi-grained (Mg) convolution modules on the model’s performance. The results of the experiments are shown in Table 4.

In terms of channel attention, the prediction accuracy significantly decreased when only joint attention was used without dual attention. The multi-granularity convolutional TCN module showed excellent performance in capturing long-term temporal dependence, thus improving the long-term prediction accuracy. Furthermore, when the channel-att or Mg module was removed, the error at 1000 ms increased by 1.9% and 4.0%, respectively, on the Human3.6M dataset, and by 2.9% and 4.0%, respectively, on the CMU-MoCap dataset. The best performance could be achieved by combining these two components. The multi-granularity model demonstrated better performance compared to the single-granularity model, especially for long prediction cycles. Additionally, the use of learnable weight parameters led to better prediction performance compared to fixed weights. This suggested that by designing a multi-granularity temporal structure, we could extract the temporal correlation between different time periods more effectively, thus improving the prediction performance.

**Effects of the Number of DA-MgTCNs:** Further, to validate the effect of multiple DA-MgTCNs in the model, we increased the number of DA-MgTCNs from 6 to 10 in step 2 and determined the prediction error and running time cost for both dataset predictions, as shown in Table 5. The experimental results showed that when 6 to 10 DA-MgTCNs were used, the predicted MPJPE decreased, while the time cost continued to increase. When 12 or 14 DA-MgTCNs were used, the prediction error remained stable at a lower level, but the time cost increased. Therefore, the use of 10 MgTCNs was chosen to achieve higher prediction accuracy and operational efficiency.

In summary, the experimental results in this paper revealed the importance of the DA-MgTCN method using the dual-attention and multi-granularity convolutional design in terms of performance improvement. Modeling joint motion trajectories using dual-attention, dual-branch spatial attention, and multi-granularity temporal convolution could provide richer and smoother temporal contextual information related to joint motion, which was conducive to adequately modeling spatial global dependencies and enabling the model to encode joint information with hidden dependencies at a distance, thus improving the overall performance of the model for both short-term and long-term motion prediction.

### 4.6. Limitations

**Figure 5:**
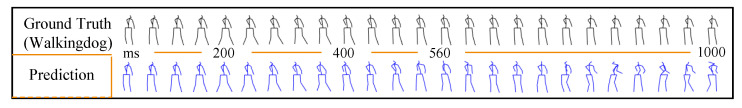
Example of the failure cases of our forecasting approach. The first line indicates the ground-truth 3D human motion. The second line, shown in blue, presents the predicted future motion.

In addition to the qualitative results presented in Figure 4, challenging cases encountered by the DA-MgTCN model were also investigated. Figure 5 illustrates an example of a predicted skeleton for the "walking a dog" action. It was evident that the last few frames did not perfectly align with the ground truth pose. This misalignment resulted from the high degree of uncertainty inherent in human motion, where a series of past poses can suggest various possible future outcomes. As a result, predicting long-term dependencies between joints and frames becomes more difficult. Furthermore, the experiments were constrained by more realistic data scenarios and experimental conditions, which may have posed challenges to our algorithm’s validation. In the future, we will consider motion prediction in more intricate scenarios to investigate novel methods for multi-grain human motion prediction in multi-domain contexts. The aim is to enhance the adaptability and performance of the model.

## 5. Conclusions

In this paper, we proposed a novel human motion prediction method leveraging dual-channel attention and multi-granularity temporal convolutional networks (DA-MgTCNs) to accurately understand and analyze human motion. Our method combined a dual-attention mechanism and a multi-granularity temporal convolutional networks model to address the challenging problem of extracting inter-joint and intra-joint spatial features. Moreover, the multi-granularity temporal convolutional networks model facilitated the design of a TCN with different convolutional kernel granularities, enabling the learning of richer multi-scale temporal information and further enhancing the performance of the model. Extensive experiments were conducted on two large-scale datasets, Human3.6M and CMU-MoCap. The experimental results demonstrated that the proposed method significantly outperformed other approaches in both short-term and long-term prediction tasks, thus validating the effectiveness of the proposed algorithm. In future work, we aim to further optimize the network structure and parameter settings and extend the application of our model to spatio-temporal prediction tasks in real-world scenarios, such as robot perception and interaction.

## Figures and Tables

**Table 1:** The choice and configuration of the relevant hyperparameters.

Hyperparameter/Config	Value
Optimizer	AdamW
Base learning rate	$3\times 10^{-3}$
Weight decay	$10^{-2}$
Optimizer momentum	$\beta_{1}=0.9,\beta_{2}=0.999$
Batch size	16
Warmup epochs	5
Epochs	60
Layer	10

**Table 2:** Prediction of 3D joint positions in Human3.6M for all actions. The best results are marked in bold.

**Time (ms)**	**80**	**160**	**320**	**400**	**560**	**1000**	**80**	**160**	**320**	**400**	**560**	**1000**
**Action**	**Walking**						**Eating**					
Res. sup. [17]	29.4	50.8	76.0	81.5	81.7	100.7	16.8	30.6	56.9	68.7	79.9	100.2
convSeq2Seq [11]	17.7	33.5	56.3	63.6	72.2	82.3	11.0	22.4	40.7	48.4	61.3	87.1
DMGNN [13]	17.3	30.7	54.6	65.2	73.4	95.8	11.0	21.4	36.2	43.9	58.1	86.7
LTD [25]	12.3	23.0	39.8	46.1	54.1	59.8	8.4	16.9	33.2	40.7	53.4	77.8
MSR [49]	12.2	22.7	38.6	45.2	52.7	63.0	8.4	17.1	33.0	40.4	52.5	77.1
Hisrep [48]	**10.0**	19.5	34.2	**39.8**	47.4	58.1	**6.4**	**14.0**	**28.7**	**36.2**	50.0	75.7
ST-DGCN [45]	10.2	19.8	34.5	40.3	48.1	56.4	7.0	15.1	30.6	38.1	51.1	76.0
Our model	10.1	**19.2**	**33.8**	40.2	**46.1**	**55.4**	7.0	14.3	30.2	38.5	**48.9**	**72.6**
**Action**	**Smoking**						**Discussion**					
Res. sup. [17]	23.0	42.6	70.1	82.7	94.8	137.4	32.9	61.2	90.9	96.2	121.3	161.7
convSeq2Seq [11]	11.6	22.8	41.3	48.9	60.0	81.7	17.1	34.5	64.8	77.6	98.1	129.3
DMGNN [13]	9.0	17.6	32.1	40.3	50.9	72.2	17.3	34.8	61.0	69.8	81.9	138.3
LTD [25]	7.9	16.2	31.9	38.9	50.7	72.6	12.5	27.4	58.5	71.7	91.6	121.5
MSR [49]	8.0	16.3	31.3	38.2	49.5	71.6	12.0	26.8	57.1	69.7	88.6	117.6
Hisrep [48]	7.0	14.9	29.9	36.4	47.6	69.5	10.2	**23.4**	**52.1**	65.4	86.6	119.8
ST-DGCN [45]	6.6	**14.1**	28.2	34.7	46.5	69.5	10.0	23.8	53.6	66.7	87.1	118.2
ours	**6.5**	14.6	**28.0**	**33.8**	**46.1**	**66.7**	**9.8**	24.2	54.5	**65.1**	**83.1**	**114.8**
**Action**	**Directions**						**Greeting**					
Res. sup. [17]	35.4	57.3	76.3	87.7	110.1	152.5	34.5	63.4	124.6	142.5	156.1	166.5
convSeq2Seq [11]	13.5	29.0	57.6	69.7	86.6	115.8	22.0	45.0	82.0	96.0	116.9	147.3
DMGNN [13]	13.1	24.6	64.7	81.9	110.1	115.8	23.3	50.3	107.3	132.1	152.5	157.7
LTD [25]	9.0	19.9	43.4	53.7	71.0	101.8	18.7	38.7	77.7	93.4	115.4	148.8
MSR [49]	8.6	19.7	43.3	53.8	71.2	100.6	16.5	37.0	77.3	93.4	116.3	147.2
Hisrep [48]	7.4	18.4	44.5	56.5	73.9	106.5	**13.7**	**30.1**	**63.8**	**78.1**	**101.9**	138.8
ST-DGCN [45]	7.2	17.6	40.9	51.5	69.3	100.4	15.2	34.1	71.6	87.1	110.2	143.5
Our model	**6.9**	**17.0**	**40.7**	**49.0**	**68.0**	**98.5**	14.6	33.3	68.5	86.4	112.0	**135.9**
**Action**	**Phoning**						**Posing**					
Res. sup. [17]	38.0	69.3	115.0	126.7	141.2	131.5	36.1	69.1	130.5	157.1	194.7	240.2
convSeq2Seq [11]	13.5	26.6	49.9	59.9	77.1	114.0	16.9	36.7	75.7	92.9	122.5	187.4
DMGNN [13]	12.5	25.8	48.1	58.3	78.9	98.6	15.3	29.3	71.5	96.7	163.9	310.1
LTD [25]	10.2	21.0	42.5	52.3	69.2	103.1	13.7	29.9	66.6	84.1	114.5	173.0
MSR citedang2021msr	10.1	20.7	41.5	51.3	68.3	104.4	12.8	29.4	67.0	85.0	116.3	174.3
Hisrep [48]	8.6	18.3	39.0	49.2	67.4	105.0	**10.2**	**24.2**	**58.5**	75.8	107.6	178.2
ST-DGCN [45]	8.3	18.3	**38.7**	48.4	65.9	102.7	10.7	25.7	60.0	76.6	106.1	164.8
Our model	**8.3**	**18.1**	39.2	**47.9**	**64.5**	**95.6**	10.4	25.4	60.5	**74.8**	**103.2**	**162.2**
**Time (ms)**	**80**	**160**	**320**	**400**	**560**	**1000**	**80**	**160**	**320**	**400**	**560**	**1000**
**Action**	**Purchases**						**Sitting**					
Res. sup. [17]	36.3	60.3	86.5	95.9	122.7	160.3	42.6	81.4	134.7	151.8	167.4	201.5
convSeq2Seq [11]	20.3	41.8	76.5	89.9	111.3	151.5	13.5	27.0	52.0	63.1	82.4	120.7
DMGNN [13]	21.4	38.7	75.7	92.7	118.6	153.8	11.9	25.1	44.6	50.2	60.1	104.9
LTD [25]	15.6	32.8	65.7	79.3	102.0	143.5	10.6	21.9	46.3	57.9	78.3	119.7
MSR [49]	14.8	32.4	66.1	79.6	101.6	139.2	10.5	22.0	46.3	57.8	78.2	120.0
Hisrep [48]	13.0	29.2	60.4	73.9	95.6	134.2	9.3	20.1	44.3	56.0	76.4	115.9
ST-DGCN [45]	**12.5**	**28.7**	60.1	73.3	95.3	133.3	8.8	19.2	42.4	53.8	74.4	116.1
Our model	12.6	29.1	**59.0**	**72.4**	**91.6**	**128.3**	**8.4**	**18.5**	**40.4**	**52.9**	**72.0**	**113.6**
**Action**	**Sitting Down**						**Taking Photo**					
Res. sup. [17]	47.3	86.0	145.8	168.9	205.3	277.6	26.1	47.6	81.4	94.7	117.0	143.2
convSeq2Seq [11]	20.7	40.6	70.4	82.7	106.5	150.3	12.7	26.0	52.1	63.6	84.4	128.1
DMGNN [13]	15.0	32.9	77.1	93.0	122.1	168.8	13.6	29.0	46.0	58.8	91.6	120.7
LTD [25]	16.1	31.1	61.5	75.5	100.0	150.2	9.9	20.9	45.0	56.6	77.4	119.8
MSR [49]	16.1	31.6	62.5	76.8	102.8	155.5	9.9	21.0	44.6	56.3	77.9	121.9
Hisrep [48]	14.9	30.7	59.1	72.0	97.0	**143.6**	8.3	18.4	40.7	51.5	72.1	**115.9**
ST-DGCN [45]	13.9	27.9	**57.4**	**71.5**	96.7	147.8	8.4	18.9	42.0	53.3	74.3	118.6
Our model	**13.8**	**27.0**	58.1	72.2	**95.7**	143.7	**8.2**	**18.1**	**40.6**	**51.2**	**70.9**	117.1
**Action**	**Waiting**						**Walking Dog**					
Res. sup. [17]	30.6	57.8	106.2	121.5	146.2	196.2	64.2	102.1	141.1	164.4	191.3	209.0
convSeq2Seq [11]	14.6	29.7	58.1	69.7	87.3	117.7	27.7	53.6	90.7	103.3	122.4	162.4
DMGNN [13]	12.2	24.2	59.6	77.5	106.0	136.7	47.1	93.3	160.1	171.2	194.0	182.3
LTD [25]	11.4	24.0	50.1	61.5	79.4	108.1	23.4	46.2	83.5	96.0	111.9	148.9
MSR [49]	10.7	23.1	48.3	59.2	76.3	106.3	20.7	42.9	80.4	93.3	111.9	148.2
Hisrep [48]	**8.7**	**19.2**	**43.4**	54.9	74.5	108.2	20.1	40.3	73.3	**86.3**	108.2	146.9
ST-DGCN [45]	8.9	20.1	43.6	54.3	72.2	103.4	18.8	39.3	73.7	86.4	**104.7**	139.8
Our model	9.2	19.9	43.6	**53.0**	**67.3**	**100.8**	**18.5**	**37.7**	**72.8**	87.6	105.8	**137.2**
**Action**	**Waiting Together**						**Average**					
Res. sup. [17]	26.8	50.1	80.2	92.2	107.6	131.1	34.7	62.0	101.1	115.5	97.6	130.5
convSeq2Seq [11]	15.3	30.4	53.1	61.2	72.0	87.4	16.6	33.3	61.4	72.7	90.7	124.2
DMGNN [13]	14.3	26.7	50.1	63.2	83.4	115.9	17.0	33.6	65.9	79.7	103.0	137.2
LTD [25]	10.5	21.0	38.5	45.2	55.0	65.6	12.7	26.1	52.3	63.5	81.6	114.3
MSR [49]	10.6	20.9	37.4	43.9	52.9	65.9	12.1	25.6	51.6	62.9	81.1	114.2
Hisrep [48]	8.9	**18.4**	35.1	41.9	52.7	64.9	10.4	22.6	47.1	58.3	77.3	112.1
ST-DGCN [45]	**8.7**	18.6	34.4	41.0	51.9	64.3	10.3	22.7	47.4	58.5	76.9	110.3
Our model	8.7	18.5	**33.5**	**40.5**	**50.8**	**61.4**	**10.2**	**22.3**	**46.9**	**57.7**	**75.1**	**106.9**

**Table 3:** Short- and long-term prediction of 3D body poses on CMU-Mocap. All results are in millimeters. The best results are marked in bold.

Time (ms)	80	160	320	400	560	1000
Res. sup. [17]	24.0	43.0	74.5	87.2	105.5	136.3
convSeq2Seq [11]	12.5	22.2	40.7	49.7	—	84.6
DMGNN [13]	13.6	24.1	47.0	58.8	77.4	112.6
LTD [25]	9.3	17.1	33.0	40.9	55.8	86.2
LPJP [44]	9.8	17.6	35.7	45.1	-	93.2
MSR [49]	8.1	15.2	30.6	38.6	53.7	83.0
ST-DGCN [45]	7.6	14.3	29.0	36.6	50.9	80.1
Our model (DA-MgTCN)	**7.5**	**14.0**	**28.1**	**34.8**	**49.0**	**77.4**

**Table 4:** Influence of the channel-attention (channel-att) and multi-grained (Mg) convolution modules on the Human3.6M and CMU-MoCap datasets. On average, the two components of our model contributed to its accuracy. The best results are marked in bold.

		**Human3.6M MPJPE (mm)**						**CMU-MoCap MPJPE (mm)**					
**Channel-att**	**MgTCN**	**80**	**160**	**320**	**400**	**560**	**1000**	**80**	**160**	**320**	**400**	**560**	**1000**
*√*		10.4	22.9	48.1	59.0	75.9	108.7	7.7	14.5	29.0	36.1	51.9	81.7
	*√*	10.5	23.1	48.0	59.4	76.8	111.0	7.9	14.6	29.2	36.7	52.3	82.6
*√*	*√*	**10.2**	**22.4**	**46.9**	**57.7**	**74.8**	**106.7**	**7.5**	**14.0**	**28.1**	**34.8**	**49.0**	**79.4**

**Table 5:** The MPJPE of our model with different numbers of DA-MgTCNs for short-term and long-term prediction on Human3.6M and CMU-MoCap. The best results are marked in bold.

**DA-**	**Human3.6M MPJPE (mm)**						**CMU-MoCap MPJPE (mm)**					
**MgTCNs**	**80**	**160**	**320**	**400**	**560**	**1000**	**80**	**160**	**320**	**400**	**560**	**1000**
6	11.5	25.0	51.9	65.6	81.1	118.0	8.4	15.5	31.7	39.5	54.3	88.3
8	10.6	23.5	49.0	61.4	78.3	111.1	7.9	14.8	29.8	37.1	52.4	82.9
10	**10.2**	**22.3**	**46.9**	**57.7**	**74.8**	106.7	**7.5**	**14.0**	**28.1**	**34.8**	**49.0**	**77.4**
12	10.3	22.7	47.9	58.6	74.6	**106.5**	7.8	13.8	28.3	35.3	50.9	78.8
14	10.3	22.8	46.8	58.0	76.3	108.1	7.6	14.3	28.9	35.6	49.2	78.4

## Data Availability

The datasets generated and/or analyzed during the current study are publicly available. The Human3.6M dataset can be accessed through the reference in [47]. The CMU-MoCap dataset is publicly available and can be accessed online at http://mocap.cs.cmu.edu/ (accessed on 13 June 2023). The use of these datasets is governed by their respective usage policies.

## Abbreviations

The following abbreviations are used in this manuscript:

DA	Dual attention
TCNs	Temporal convolutional networks
MgTCN	Multi-granularity TCN
GNNs	Graph neural networks
